# 10-(Prop-2-yn-1-yl)-2,7-diaza­phenothia­zine^1^

**DOI:** 10.1107/S1600536812018879

**Published:** 2012-05-02

**Authors:** Beata Morak-Młodawska, Kinga Suwińska, Krystian Pluta, Małgorzata Jeleń

**Affiliations:** a Department of Organic Chemistry, The Medical University of Silesia, ul. Jagiellońska 4, 41-200 Sosnowiec, Poland; b Institute of Physical Chemistry, Polish Academy of Sciences, ul. Kasprzaka 44/52, 01-224 Warsaw, Poland; c Faculty of Biology and Environmental Sciences, Cardinal Stefan Wyszynski University, ul. Wóycickiego 1/3, 01 938 Warszawa, Poland

## Abstract

In the title mol­ecule [systematic name: 10-(prop-2-yn-1-yl)dipyrido[3,4-*b*:3′,4′-*e*][1,4]thia­zine], C_13_H_9_N_3_S, the dihedral angle between the two pyridine rings is 146.33 (7)° and the angle between two halves of the thia­zine ring is 138.84 (8)°, resulting in a butterfly shape for the tricyclic system. The central thia­zine ring adopts a boat conformation, with the 2-propynyl substituent at the thia­zine N atom located in a pseudo-equatorial position and oriented to the concave side of the diaza­phenothia­zine system. In the crystal, mol­ecules are arranged *via* π–π inter­actions between the pyridine rings [centroid–centroid distances = 3.838 (1) and 3.845 (1) Å] into stacks extending along [001]. There are C—H⋯C and C—H⋯N inter­actions between mol­ecules of neighbouring stacks.

## Related literature

For recent literature on the biological activity of phenothia­zines, see: Aaron *et al.* (2009 ▶); Pluta *et al.* (2011 ▶). For the structure of 10-(2-propyn­yl)phenothia­zine and its transformations into anti­cancer derivatives, see: Bisi *et al.* (2008 ▶). For the synthesis and the anti­cancer and immunosuppressive activity of 2,7-diaza­phenothia­zines, see: Morak-Młodawska & Pluta (2009 ▶); Zimecki *et al.* (2009 ▶); Pluta *et al.* (2010 ▶). For planar and folded structures of the 2,7-diaza­phenothia­zine system, see: Morak *et al.* (2002 ▶); Morak-Młodawska *et al.* (2010 ▶). For alkyl­ation of aza­phenothia­zines, see: Pluta *et al.* (2009 ▶).
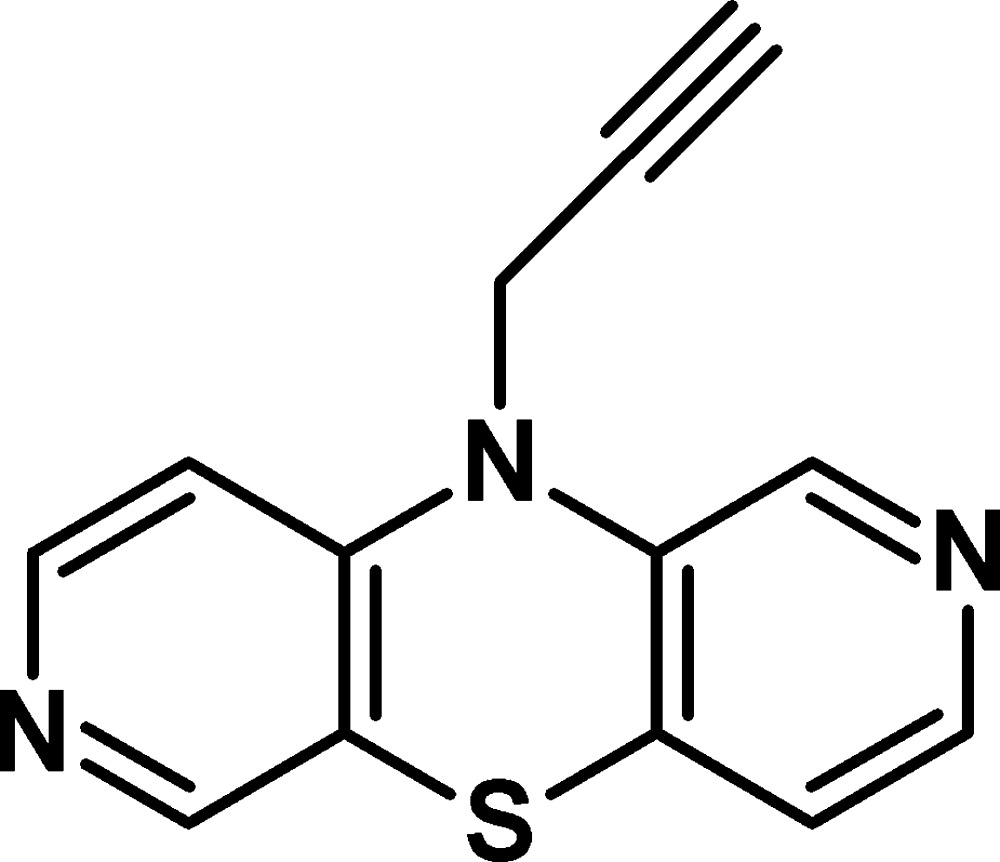


## Experimental

### 

#### Crystal data

C_13_H_9_N_3_S
*M* *_r_* = 239.29
Monoclinic, 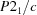

*a* = 14.1150 (9) Å
*b* = 10.1909 (6) Å
*c* = 7.6749 (5) Å
β = 104.212 (3)°
*V* = 1070.20 (12) Å^3^
*Z* = 4
Mo *K*α radiation
μ = 0.28 mm^−1^
*T* = 100 K
0.60 × 0.50 × 0.35 mm

#### Data collection

Nonius KappaCCD diffractometer upgraded with APEXII detector
7015 measured reflections
2407 independent reflections
2011 reflections with *I* > 2σ(*I*)
*R*_int_ = 0.041

#### Refinement

*R*[*F* ^2^ > 2σ(*F* ^2^)] = 0.049
*wR*(*F* ^2^) = 0.114
*S* = 1.11
2407 reflections
154 parameters
H-atom parameters constrained
Δρ_max_ = 0.45 e Å^−3^
Δρ_min_ = −0.35 e Å^−3^

### 

Data collection: *COLLECT* (Nonius, 1998 ▶); cell refinement: *DENZO* and *SCALEPACK* (Otwinowski & Minor, 1997 ▶); data reduction: *DENZO* and *SCALEPACK*; program(s) used to solve structure: *SHELXS97* (Sheldrick, 2008 ▶); program(s) used to refine structure: *SHELXL97* (Sheldrick, 2008 ▶); molecular graphics: *ORTEPIII* (Burnett & Johnson, 1996 ▶) and *Mercury* (Macrae *et al.*, 2008 ▶); software used to prepare material for publication: *publCIF* (Westrip, 2010 ▶).

## Supplementary Material

Crystal structure: contains datablock(s) I, global. DOI: 10.1107/S1600536812018879/gk2475sup1.cif

Structure factors: contains datablock(s) I. DOI: 10.1107/S1600536812018879/gk2475Isup2.hkl

Supplementary material file. DOI: 10.1107/S1600536812018879/gk2475Isup3.cml

Additional supplementary materials:  crystallographic information; 3D view; checkCIF report

## Figures and Tables

**Table 1:** Hydrogen-bond geometry (Å, °)

*D*—H⋯*A*	*D*—H	H⋯*A*	*D*⋯*A*	*D*—H⋯*A*
C4—H4⋯N2^i^	0.95	2.62	3.457 (3)	147
C13—H13⋯C11^ii^	0.95	2.78	3.677 (3)	159
C13—H13⋯C12^ii^	0.95	2.78	3.686 (3)	161
C3—H3⋯C13^i^	0.95	2.78	3.662 (3)	155
C8—H8⋯C13^iii^	0.95	2.69	3.407 (3)	133

Symmetry codes: (i) 
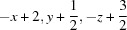
; (ii) 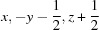; (iii) 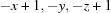.

**Fig. 1.**
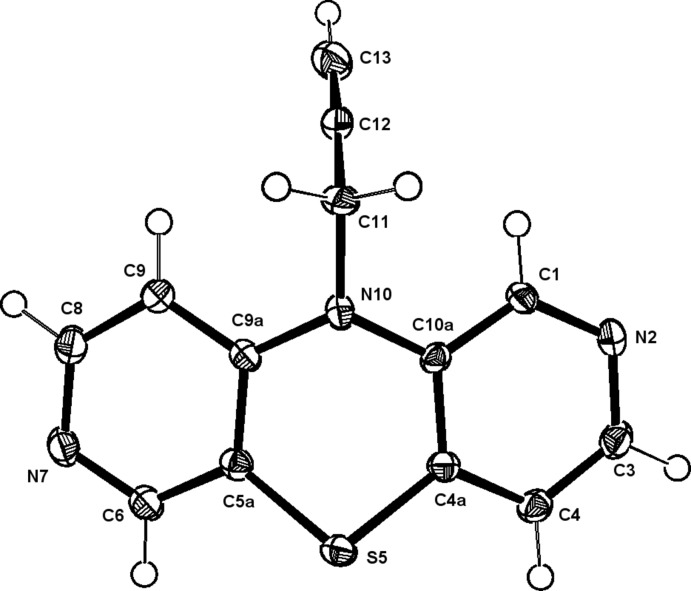
ORTEP drawing with displacement ellipsoids shown at the 50% probability level.

**Fig. 2.**
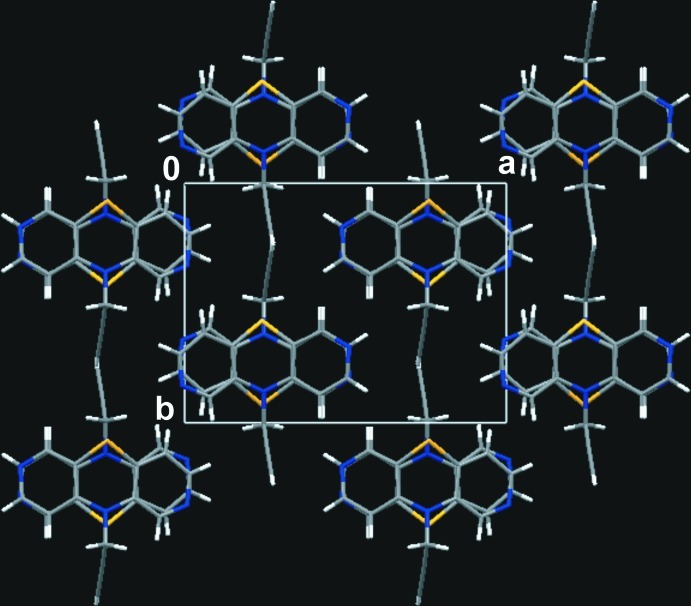
Crystal packing shown along the c axis.

## supplementary crystallographic information

### Comment

Phenothiazines exhibit not only recognized neuroleptic, antihistaminic and antitussive activities but recently also anticancer, antibacterial and reversal multidrug resistance [Aaron *et al.*, (2009); Pluta *et al.*, (2011)]. The modifications of the phenothiazine structures are mainly directed into the introduction of new pharmacophoric substituents at the thiazine nitrogen atom and the substitution of the benzene ring with an azine ring (Pluta *et al.*, 2009, 2011). Synthesis of substituted 10-(2-propynyl)phenothiazines and their transformations into various aminobutynyl derivatives of anticancer and multidrug resistance reverting activities was reported by Bisi *et al.* (2008). We modified the phenothiazine structure *via* the substitution of the benzene ring with the pyridine ring to form 2,7-diazaphenothiazines (Morak-Młodawska & Pluta, 2009) possessing anticancer and immunosuppressive activities (Zimecki *et al.*, 2009; Pluta *et al.* 2010). Alkylation of azaphenothiazines proceeds at the thiazine and/or the azine nitrogen atoms, depending on the reaction conditions (Pluta *et al.*, 2009). *N*-Alkylation of 10*H*-2,7-diazaphenothiazine led to both types of the products showing planar and folded 2,7-diazaphenothiazine ring system (Morak-Młodawska *et al.*, 2010). 10*H*-2,7-Diazaphenothiazine was transformed into the title compound, C_13_H_9_N_3_S, a convenient substrate to other 2,7-diazaphenothiazine derivatives using aminomethylation or 1,3-dipolar cycloaddition. The X-ray study showed the propynyl group to be attached to the thiazine nitrogen atom. In the molecule, the butterfly angle between the two pyridine rings is 146.33 (7)° and the angle between two halves of the thiazine ring is 138.84 (8)°. The 2-propynyl substituent is in a pseudo-equatorial position with the angle S5···N10–C11 of 163.8 (2)° and directed to the concave side of the diazaphenothiazine system with the angle between the N10/C11/C12/C13 and C4a/C5a/C9a/C10a planes of 86.3 (1)°. The thiazine nitrogen atom shows pyramidality as the sum of the C–N10–C bond angles is 356.1 (1)°. Hydrogen bond C4–H4···N2 (Table 1) results in one-dimensional polymeric chain parallel to the *b* axis. Acidic hydrogen atom H13 is in close contact to C11 and C12 atoms of the propynyl substituent (both H···C distances equal to 2.78 Å). This suggests, that H13 is involved in C–H···C interactions to these two carbon atoms rather than in the C–H···π interaction to the π system of the triple C12≡C13 bond (H13···center_C12__≡__C13_ distance of 2.96 Å ). Additionally, the C12≡ C13 bond π electrons interact with two aromatic H atoms (H3 and H8) of two other adjacent molecules with short C–H···C intermolecular contacts (less than the sum of van der Waals radii) between H3 and H8, and C13 (see Table 1). On the basis of these interactions a three-dimensional network is formed. Molecules π-stack along the *c* axis. Aromatic rings N2/C1/C10*a*/C4a/C4/C3 π-stack with centroid-to-centroid distance of 3.845 (1) Å, similarly, for rings N7/C6/C5A/C9A/C9/C8 the centroid-to-centroid distance is 3.838 (1) Å (see Figure 2).

### Experimental

To a suspension of 10*H*-2,7-diazaphenothiazine (100 mg, 0.5 mmol) in 5 ml DMF potassium *tert*-butoxide (80 mg, 0.72 mmol) was added. The mixture was stirred at room temperature for 1 h. Then a solution of propargyl bromide (80 mg, 0.64 mmol) in toluene was added dropwise. The solution was stirred at room temperature for 24 h and poured into water (15 ml), extracted with methylene chloride (15 ml), dried with Na_2_SO_4_ and evaporated to the brown oil. The residue was purified by column chromatography (silica gel, CHCl_3_) to yield 10-(2-propynyl)-2,7-diazaphenothiazine (72 mg, 60%), mp. 149–150°C. ^1^H NMR in CDCl_3_: δ 2.57 (t, J = 2.5 Hz, 1H), 4.54(d, J = 2.5 Hz, 2H), 7.14 (m, 2H, H-9, H-4), 8.12 (s, 1H, H-1), 8.22 (d, J = 5.5 Hz, H-3), 8.35 (d, J = 5.5 Hz, H-8), 8.40 (s, 1H, H-6). FAB MS: 240 (*M*+H, 100), 201 (M—CH_2_CCH+1, 45).

### Refinement

All H atoms in the were treated as riding atoms in geometrically idealized positions, with C–H distances of 0.95 (aromatic and acetylene) or 0.99 Å (methylene), and with *U*_iso_(H) = 1.2*U*_eq_(C).

### Crystal data

**Table:**

C_13_H_9_N_3_S	*F*(000) = 496
*M**_r_* = 239.29	*D*_x_ = 1.485 Mg m^−^^3^
Monoclinic, *P*2_1_/*c*	Mo *K*α radiation, λ = 0.71073 Å
Hall symbol: -P 2ybc	Cell parameters from 2229 reflections
*a* = 14.1150 (9) Å	θ = 2.5–27.5°
*b* = 10.1909 (6) Å	µ = 0.28 mm^−^^1^
*c* = 7.6749 (5) Å	*T* = 100 K
β = 104.212 (3)°	Block, yellow
*V* = 1070.20 (12) Å^3^	0.60 × 0.50 × 0.35 mm
*Z* = 4	

### Data collection

**Table:**

Nonius KappaCCD diffractometer upgraded with APEXII detector	2011 reflections with *I* > 2σ(*I*)
Radiation source: fine-focus sealed tube	*R*_int_ = 0.041
Graphite monochromator	θ_max_ = 27.5°, θ_min_ = 3.4°
Detector resolution: 8.3 pixels mm^-1^	*h* = −18→18
ω scan	*k* = −12→13
7015 measured reflections	*l* = −9→9
2407 independent reflections	

### Refinement

**Table:**

Refinement on *F*^2^	Primary atom site location: structure-invariant direct methods
Least-squares matrix: full	Secondary atom site location: difference Fourier map
*R*[*F*^2^ > 2σ(*F*^2^)] = 0.049	Hydrogen site location: inferred from neighbouring sites
*wR*(*F*^2^) = 0.114	H-atom parameters constrained
*S* = 1.11	*w* = 1/[σ^2^(*F*_o_^2^) + (0.0285*P*)^2^ + 1.4885*P*] where *P* = (*F*_o_^2^ + 2*F*_c_^2^)/3
2407 reflections	(Δ/σ)_max_ = 0.001
154 parameters	Δρ_max_ = 0.45 e Å^−^^3^
0 restraints	Δρ_min_ = −0.35 e Å^−^^3^

### Special details

**Table:**

Geometry. All e.s.d.'s (except the e.s.d. in the dihedral angle between two l.s. planes) are estimated using the full covariance matrix. The cell e.s.d.'s are taken into account individually in the estimation of e.s.d.'s in distances, angles and torsion angles; correlations between e.s.d.'s in cell parameters are only used when they are defined by crystal symmetry. An approximate (isotropic) treatment of cell e.s.d.'s is used for estimating e.s.d.'s involving l.s. planes.
Refinement. Refinement of *F*^2^ against ALL reflections. The weighted *R*-factor *wR* and goodness of fit *S* are based on *F*^2^, conventional *R*-factors *R* are based on *F*, with *F* set to zero for negative *F*^2^. The threshold expression of *F*^2^ > 2σ(*F*^2^) is used only for calculating *R*-factors(gt) *etc*. and is not relevant to the choice of reflections for refinement. *R*-factors based on *F*^2^ are statistically about twice as large as those based on *F*, and *R*- factors based on ALL data will be even larger.

### Fractional atomic coordinates and isotropic or equivalent isotropic displacement parameters (Å^2^)

**Table:**

	*x*	*y*	*z*	*U*_iso_*/*U*_eq_	
C1	0.92188 (15)	0.1074 (2)	0.5605 (3)	0.0172 (4)	
H1	0.9145	0.0148	0.5522	0.021*	
C3	1.01573 (17)	0.2865 (2)	0.6685 (3)	0.0218 (5)	
H3	1.0762	0.3225	0.7335	0.026*	
C4	0.94013 (16)	0.3721 (2)	0.5945 (3)	0.0195 (5)	
H4	0.9479	0.4640	0.6135	0.023*	
C4a	0.85325 (16)	0.3212 (2)	0.4927 (3)	0.0159 (4)	
C5a	0.65940 (16)	0.3263 (2)	0.3914 (3)	0.0164 (4)	
C6	0.57174 (16)	0.3834 (2)	0.4013 (3)	0.0197 (5)	
H6	0.5703	0.4759	0.4161	0.024*	
C8	0.49541 (17)	0.1852 (2)	0.3756 (3)	0.0226 (5)	
H8	0.4377	0.1351	0.3665	0.027*	
C9	0.58017 (16)	0.1180 (2)	0.3718 (3)	0.0192 (5)	
H9	0.5801	0.0249	0.3640	0.023*	
C9a	0.66564 (15)	0.1889 (2)	0.3797 (3)	0.0154 (4)	
C10a	0.84271 (15)	0.1850 (2)	0.4757 (3)	0.0147 (4)	
C11	0.75374 (17)	−0.0072 (2)	0.3179 (3)	0.0182 (5)	
H11a	0.8152	−0.0251	0.2819	0.022*	
H11b	0.6992	−0.0198	0.2102	0.022*	
C12	0.74397 (16)	−0.1054 (2)	0.4530 (3)	0.0194 (5)	
C13	0.73370 (18)	−0.1884 (3)	0.5542 (4)	0.0284 (6)	
H13	0.7254	−0.2552	0.6356	0.034*	
S5	0.75938 (4)	0.42507 (5)	0.37659 (8)	0.01880 (16)	
N2	1.00796 (13)	0.15579 (19)	0.6532 (3)	0.0204 (4)	
N7	0.48877 (14)	0.3163 (2)	0.3912 (3)	0.0226 (4)	
N10	0.75434 (13)	0.12936 (18)	0.3721 (2)	0.0158 (4)	

### Atomic displacement parameters (Å^2^)

**Table:**

	*U*^11^	*U*^22^	*U*^33^	*U*^12^	*U*^13^	*U*^23^
C1	0.0160 (10)	0.0151 (10)	0.0217 (11)	0.0027 (8)	0.0071 (9)	0.0011 (8)
C3	0.0170 (11)	0.0228 (12)	0.0250 (12)	−0.0042 (10)	0.0041 (9)	−0.0051 (10)
C4	0.0194 (11)	0.0165 (10)	0.0244 (12)	−0.0023 (9)	0.0085 (9)	−0.0050 (9)
C4a	0.0165 (10)	0.0147 (10)	0.0178 (11)	0.0005 (8)	0.0070 (8)	0.0003 (8)
C5a	0.0176 (10)	0.0166 (10)	0.0142 (10)	0.0009 (8)	0.0025 (8)	0.0013 (8)
C6	0.0195 (11)	0.0186 (10)	0.0197 (11)	0.0028 (9)	0.0023 (9)	−0.0007 (9)
C8	0.0168 (11)	0.0245 (12)	0.0250 (12)	−0.0011 (9)	0.0024 (9)	−0.0024 (10)
C9	0.0175 (10)	0.0170 (10)	0.0217 (11)	−0.0004 (9)	0.0022 (9)	−0.0013 (9)
C9a	0.0155 (10)	0.0156 (10)	0.0143 (10)	0.0027 (8)	0.0022 (8)	−0.0005 (8)
C10a	0.0141 (10)	0.0144 (10)	0.0169 (11)	−0.0017 (8)	0.0064 (8)	0.0000 (8)
C11	0.0199 (11)	0.0136 (10)	0.0218 (11)	0.0011 (9)	0.0064 (9)	−0.0028 (9)
C12	0.0149 (10)	0.0177 (10)	0.0250 (12)	0.0000 (9)	0.0037 (9)	−0.0034 (9)
C13	0.0256 (13)	0.0242 (12)	0.0360 (15)	0.0034 (10)	0.0086 (11)	0.0067 (11)
S5	0.0187 (3)	0.0143 (3)	0.0241 (3)	0.0015 (2)	0.0066 (2)	0.0035 (2)
N2	0.0131 (9)	0.0226 (10)	0.0248 (10)	0.0021 (8)	0.0033 (7)	−0.0002 (8)
N7	0.0164 (9)	0.0244 (10)	0.0254 (11)	0.0039 (8)	0.0020 (8)	−0.0014 (8)
N10	0.0119 (8)	0.0185 (9)	0.0167 (9)	0.0014 (7)	0.0030 (7)	−0.0005 (7)

### Geometric parameters (Å, º)

**Table:**

C1—N2	1.342 (3)	C6—H6	0.9500
C1—C10a	1.393 (3)	C8—N7	1.347 (3)
C1—H1	0.9500	C8—C9	1.385 (3)
C3—N2	1.339 (3)	C8—H8	0.9500
C3—C4	1.387 (3)	C9—C9a	1.395 (3)
C3—H3	0.9500	C9—H9	0.9500
C4—C4a	1.383 (3)	C9a—N10	1.405 (3)
C4—H4	0.9500	C10a—N10	1.422 (3)
C4a—C10a	1.398 (3)	C11—N10	1.452 (3)
C4a—S5	1.758 (2)	C11—C12	1.471 (3)
C5a—C6	1.386 (3)	C11—H11a	0.9900
C5a—C9a	1.407 (3)	C11—H11b	0.9900
C5a—S5	1.760 (2)	C12—C13	1.181 (3)
C6—N7	1.342 (3)	C13—H13	0.9500
			
N2—C1—C10a	123.9 (2)	C8—C9—H9	120.5
N2—C1—H1	118.1	C9a—C9—H9	120.5
C10a—C1—H1	118.1	C9—C9a—N10	122.98 (19)
N2—C3—C4	123.5 (2)	C9—C9a—C5a	116.82 (19)
N2—C3—H3	118.3	N10—C9a—C5a	120.18 (19)
C4—C3—H3	118.3	C1—C10a—C4a	117.7 (2)
C4a—C4—C3	118.8 (2)	C1—C10a—N10	121.87 (19)
C4a—C4—H4	120.6	C4a—C10a—N10	120.42 (19)
C3—C4—H4	120.6	N10—C11—C12	116.42 (19)
C4—C4a—C10a	119.0 (2)	N10—C11—H11a	108.2
C4—C4a—S5	120.91 (17)	C12—C11—H11a	108.2
C10a—C4a—S5	120.04 (17)	N10—C11—H11b	108.2
C6—C5a—C9a	119.5 (2)	C12—C11—H11b	108.2
C6—C5a—S5	120.26 (17)	H11a—C11—H11b	107.3
C9a—C5a—S5	120.09 (16)	C13—C12—C11	176.5 (3)
N7—C6—C5a	124.1 (2)	C12—C13—H13	180.0
N7—C6—H6	117.9	C4a—S5—C5a	98.00 (10)
C5a—C6—H6	117.9	C3—N2—C1	117.1 (2)
N7—C8—C9	124.9 (2)	C6—N7—C8	115.6 (2)
N7—C8—H8	117.6	C9a—N10—C10a	118.21 (18)
C9—C8—H8	117.6	C9a—N10—C11	118.89 (18)
C8—C9—C9a	119.0 (2)	C10a—N10—C11	119.00 (18)
			
N2—C3—C4—C4a	−2.9 (4)	C4—C4a—S5—C5a	−148.01 (19)
C3—C4—C4a—C10a	3.3 (3)	C10a—C4a—S5—C5a	35.26 (19)
C3—C4—C4a—S5	−173.43 (17)	C6—C5a—S5—C4a	148.16 (19)
C9a—C5a—C6—N7	−3.6 (4)	C9a—C5a—S5—C4a	−36.6 (2)
S5—C5a—C6—N7	171.68 (18)	C4—C3—N2—C1	0.1 (3)
N7—C8—C9—C9a	−1.8 (4)	C10a—C1—N2—C3	2.2 (3)
C8—C9—C9a—N10	−178.4 (2)	C5a—C6—N7—C8	1.9 (3)
C8—C9—C9a—C5a	0.1 (3)	C9—C8—N7—C6	0.8 (4)
C6—C5a—C9a—C9	2.4 (3)	C9—C9a—N10—C10a	−144.8 (2)
S5—C5a—C9a—C9	−172.88 (17)	C5a—C9a—N10—C10a	36.8 (3)
C6—C5a—C9a—N10	−179.1 (2)	C9—C9a—N10—C11	12.8 (3)
S5—C5a—C9a—N10	5.6 (3)	C5a—C9a—N10—C11	−165.6 (2)
N2—C1—C10a—C4a	−1.7 (3)	C1—C10a—N10—C9a	142.9 (2)
N2—C1—C10a—N10	177.10 (19)	C4a—C10a—N10—C9a	−38.3 (3)
C4—C4a—C10a—C1	−1.2 (3)	C1—C10a—N10—C11	−14.6 (3)
S5—C4a—C10a—C1	175.63 (16)	C4a—C10a—N10—C11	164.16 (19)
C4—C4a—C10a—N10	179.98 (19)	C12—C11—N10—C9a	−76.0 (3)
S5—C4a—C10a—N10	−3.2 (3)	C12—C11—N10—C10a	81.3 (2)

### Hydrogen-bond geometry (Å, º)

**Table:**

*D*—H···*A*	*D*—H	H···*A*	*D*···*A*	*D*—H···*A*
C4—H4···N2^i^	0.95	2.62	3.457 (3)	147
C13—H13···C11^ii^	0.95	2.78	3.677 (3)	159
C13—H13···C12^ii^	0.95	2.78	3.686 (3)	161
C3—H3···C13^i^	0.95	2.78	3.662 (3)	155
C8—H8···C13^iii^	0.95	2.69	3.407 (3)	133

Symmetry codes: (i) −*x*+2, *y*+1/2, −*z*+3/2; (ii) *x*, −*y*−1/2, *z*+1/2; (iii) −*x*+1, −*y*, −*z*+1.
